# Novel approach to determine unbalanced current circuit on Nigerian 330kV transmission grid for reliability and security enhancement

**DOI:** 10.1016/j.heliyon.2021.e07563

**Published:** 2021-07-15

**Authors:** Ademola Abdulkareem, A. Adesanya, A.F. Agbetuyi, A.S. Alayande

**Affiliations:** a Department of Electrical and Information Engineering, Covenant University, Nigeria; b Department of Electrical and Electronics Engineering, University of Lagos, Nigeria

**Keywords:** Novel technique, Optimize current, Symmetrical component algorithm, Numerical simulation, Line current spectrum, Unbalanced current circuit, Critical lines losses, Quad bundle parameter

## Abstract

The present Nigerian transmission network is faced with the difficulty of evacuating and dispatching reliable and quality electricity supply and simultaneously maintaining an operational standard of security to prevent any collapses. Therefore, this study developed a novel technique to optimize electrical current flow to provide in-depth research and analysis of current flowing in the transmission network circuit prone to danger during short-circuit faults. The research methodology involved the generation of unbalanced short-circuit calculations at every single node of the three-phase network using the symmetrical component method. Numerical simulation of different types of unbalanced short-circuit fault into the entire 330kV transmission network using unbalanced fault algorithms written in a flexible MATLAB program environment is also performed on every bus. The influence of these short-circuit faults is examined on the generated spectrum of line current magnitude. This study then generates a series of unbalanced current circuit and line losses analysis that unveils the different scenarios regarding existing network performance. The method adopted is promising. It established the most critical lines (about 20) with high unbalanced current magnitudes and high line losses during the disturbance. Based on the result analysis, four (quad) bundles of conductors is designed as a proposed modification to the upgrade of all critical double circuit lines and the conversion of single critical lines on the 330kV transmission network to improve the power transfer capability and also meet the future transmission network development plan. Furthermore, recommendations that are considered desirable in this study are proffered to ensure acceptable power quality and security in the network.

## 1 Introduction

The rapid growth of the world population has indeed increased energy demand significantly. Therefore, it has become essential to operating energy delivered to transmission lines at maximum efficiency [1]. Unfortunately, the existing Nigerian 330-kV transmission lines network connects all power plants and load centres in all parts of the country; it is predominantly characterized by high power loss, estimated between 9.2% -10% of the total losses the network [2, 3]. In Nigeria, the overall transmission and distribution losses are in the mega range of 25–40% [4,5]. The annual technical energy losses on the 330kV power line for 2015 as estimated by ref. [6] due to the low, medium, and high-power losses were respectively found to be 443.45GWH, 976.895GWH, and 2231.230GWH, amounting to N8.4 billion, N18.6 billion an5 N42.4 billion, respectively. Thus, there is a need to increase the 330-kV Nigerian transmission network's efficiency to successfully and efficiently transmit electricity to all parts of the country. The improvement in the present state of the system becomes essential because the construction of new generating stations and transmission requires considerable capital [7], which the Federal Government of Nigeria cannot afford at present in the course of COVID 19. For instance, the proposed plan was developed for transmission network expansion in 2004 [8]. Adding more loops system to the network to achieve higher efficiency and reliability has not been fully implemented until the project cost. Besides extensive research on the potential of renewable energy sources in Nigeria, its development has not attracted attention, unlike developed and emerging countries such as Germany, the USA, UK, and China [9]. Meanwhile, the various efforts to save the power shortfall in Nigeria have not produced the expected result [10]. Therefore, generation, transmission, and distribution infrastructures must remain balanced in the entire network for the consumer to have access to a reliable and adequate electric power supply. Presently, the privatization of the previously government-owned power generation and distribution companies has failed and did not yield the expected result of increased generation and delivering more power at distribution [11, 12, 13]. Thus, the privatized system's success still depends on the transmission system's efficacy, which reveals that if the generation sector is to run at total production, the Nigeria 330kV transmission grid will not have the capacity to evacuate the generated power [14].

Globally, many major blackouts caused by power system insecurity have been traced to fault on power lines. For example, the North American transmission system's interruption, occurring in 2003, interrupted supply for over 50 million people [15]. The two largest blackouts in the Swedish transmission system, occurring in 1983 and 2003 [16], have been estimated to 1100 MSEK and 334 MSEK, respectively [17]. In Zambia, the nationwide blackout commenced by spurious tripping on the only 330-kV transmission and a collapse of the system voltage [18]. Moreover, the transmission grid system in Nigeria is prevalently structured by radial, fragile, and very long power lines, part of which risk partial or total system collapse in the occurrence of major fault [19, 20, 21]. These lines involve Birnin Kebbi-Kanji (310KM), Benin-Ikeja West (280KM), Jos-Gombe (265KM), Ikeja West-Oshogbo (252KM), Oshogbo-Benin (251KM), and Jebba TS-Shiroro (244KM). The Nigeria 330kV transmission system is very much exposed to different types of faults ranging from balanced and unbalanced faults occurring at various systems [22, 23]. In Nigeria's 330-kV transmission system, the frequency of unbalance faults is highest at the power lines. The causes of a grid failure as analyzed from 1987 to 2009 revealed 78.6% transmission faults and 21.4% faults from the generating units out of 276 grid-failures experienced in this period [24]. Moreover, between January and June 2012, nine (9) full and five (5) partial system collapse occurred [25]. Besides, the Nigerian power grid has experienced sixteen (16) complete and five (5) partial collapses in the first six (6) months in 2016 alone [26]. The Alaoji-Afam transmission station tripped nine (9) times on fault between 06/05/2015 and 01/09/2017, which revealed the influence of fault resistance on the relay operation [27, 28, 28]. References [29, 30] researched power outages and insecurity in the Nigerian transmission grid; the outcome results provide interesting information on the infirmity influence of fault occurrence. The literature on load-flow iterative solutions analysis performed on Nigeria 330-kV network (test system) is almost in flux, but a regular problem facing researchers for so long is finding the solution to the inaccuracy in the correct assessment of the flow of active and reactive power and line losses [31, 32], for the security and reliability enhancement analysis. In most cases, the magnitude, as well as the location of the voltage, violated buses are fairly determined, but there are discrepancies in the values of active and reactive powers and the overall losses [33].

Therefore, this study provides in-depth knowledge of the flow of current in the network to solve the challenging problems inherent in designing the present and future power systems to deliver increasing electrical energy safely and in a sustainable economic manner. The more the power that flows through the power lines, the more will be the current that flows and consequently the power quality will decline. This study's uniqueness is in the novel technique developed to optimize the flow of electrical current, a significant quantity, in the network, which provides insight into the contingency analysis of the transmission lines' security dispatch operation. The approach involves the numerical simulation of various aspects of unbalance faults; single-line to the ground (SLG), line-line (LL), double line to ground (DLG) into each line of the three-phase network. The study's generated results showed that most of the lines (about 20) have high levels of unbalanced current magnitudes. The analysis covers all the 33 lines for the simulated 28 buses and categorized into four scenarios based on their characteristics, as revealed in this study, making it easy to determine and analyse the critical lines or weak areas with high losses. Further analysis of the result of the unbalanced current circuit and power line losses leads to the proposed reconductoring of quad bundles conductor design. Recommendations are made for the critical weak lines by supplying relevant information to improve the network's weak lines and future expansion planning.

## 2 Materials and methods

The data collected from PHCN includes the 330-kV transmission network in Nigeria, with particular attention to the schematic diagram of the entire single-line diagram, line data, and bus data. The data employed in the analysis are generated by the simulation of different types of unbalanced short-circuit faults into the network using unbalanced fault algorithms written in a flexible MATLAB program environment at every bus to examine their effects line current. The systematic, theoretical analysis of the methods applied and principles associated with the study areas discussed.

### 2.1 Test system for unbalance-current circuit analysis

**Figure 1:**
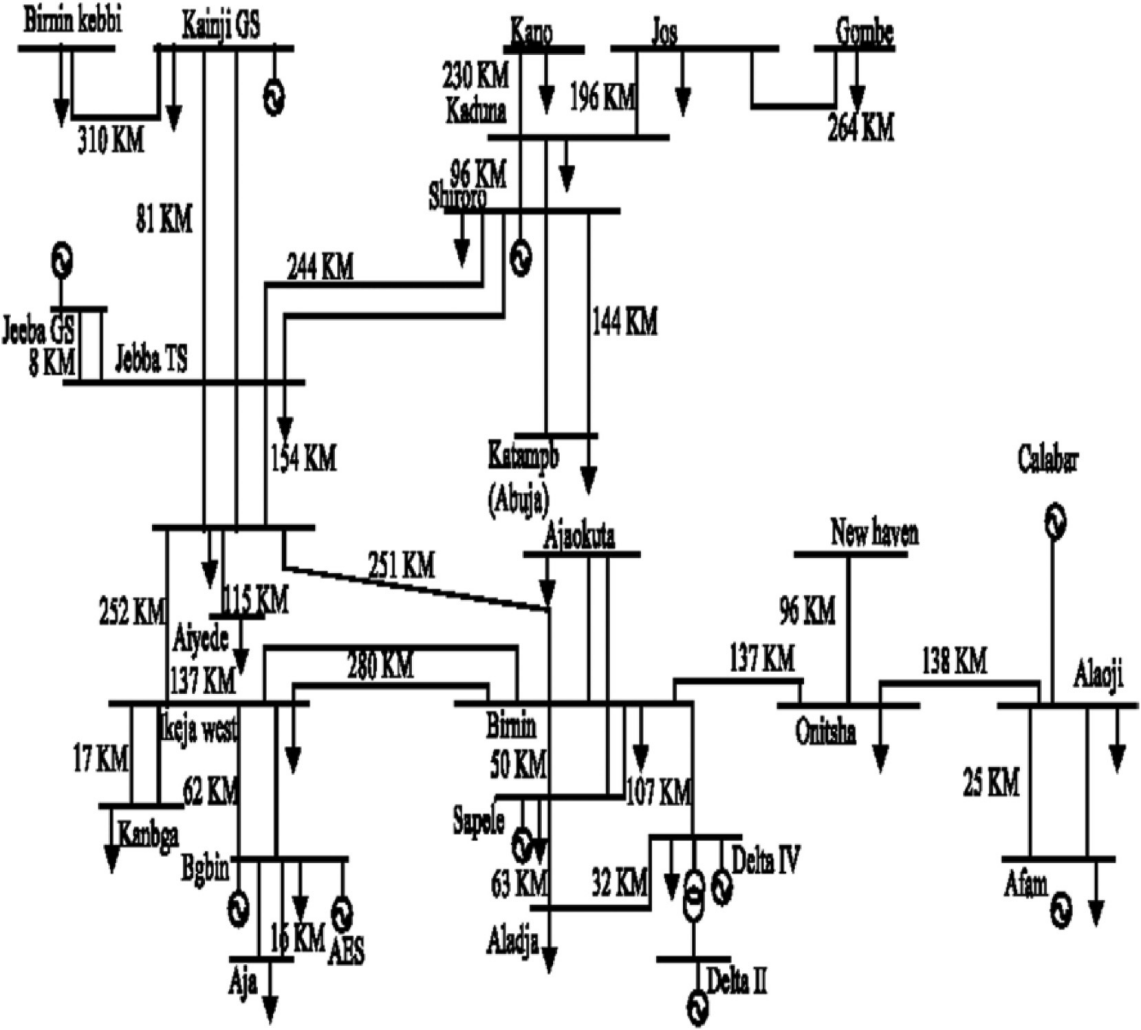
Single-line diagram of Nigeria 330kV Power System Network.

In this study, the Nigerian power network is used as the test system. The one-line diagram of the existing 28 bus and thirty-three (33) branches of the 330-kV Nigerian power system is given in Figure 1. The number of circuits and length of the interconnected lines with positive and zero sequence impedances data, as shown in Table 1 describes the transmission lines used in this study's analysis.

**Table 1:** Nigeria 330-kV transmission lines data with positive and zero sequence impedances.

Bus no/Bus name		Bus no/Bus name		Length	Circuit Type	R1	X1	R0	X0
From		To		(km)					
1	Kainji	2	Birnin-Kebbi	310	Single	0.0029	0.0246	0.0205	0.0073
1	Kainji	3	Jebba TS	81	Double	0.0111	0.0942	0.0786	0.28
4	Jebba (GS)	3	Jebba (TS)	8	Double	0.0003	0.0022	0.002	0.0073
5	Osogbo	3	Jebba (TS)	157	3 x Single	0.0056	0.0477	0.0398	0.142
5	Osogbo	6	Ayede	115	Single	0.0041	0.0349	0.0291	0.104
7	Papalanto	6	Ayede	60	Single	0.0022	0.0182	0.0152	0.0543
8	Ikeja-West	5	Oshogbo	252	Single	0.0049	0.0416	0.0341	0.214
8	Ikeja-West	6	Ayede	137	Single	0.001	0.0091	0.0076	0.0271
8	Ikeja-West	7	Papanlato	30	Single	0.0049	0.0416	0.0341	0.214
8	Ikeja-West	9	Akangba	18	Double	0.0022	0.0172	0.0155	0.0567
10	Egbin	8	Ikeja-West	62	Double	0.0022	0.0188	0.0157	0.0561
10	Egbin	13	Benin	218	Single	0.0022	0.0172	0.0155	0.0567
10	Egbin	11	Aja	14	Double	0.0057	0.486	0.0406	0.1447
12	Omotosho	8	Ikeja-West	160	Single	0.0078	0.0663	0.0553	0.1972
12	Omotosho	13	Benin	120	Single	0.01	0.0779	0.0705	0.256
13	Benin	5	Oshogbo	251	Single	0.0043	0.0365	0.0304	0.1085
13	Benin	8	Ikeja-West	280	Double	0.0089	0.0763	0.0636	0.227
13	Benin	18	Onitsha	137	Single	0.007	0.0593	0.0494	0.1764
14	Ajaokuta	13	Benin	195	Double	0.0018	0.0139	0.0126	0.0458
15	Sapele	13	Benin	50	Double	0.0023	0.019	0.016	0.057
15	Sapele	17	Aladja	63	Single	0.0023	0.019	0.016	0.057
16	Delta	13	Benin	107	Single	0.0023	0.019	0.016	0.057
16	Delta	17	Aladja	32	Single	0.0049	0.0416	0.0347	0.124
18	Onitsha	20	New Haven	96	Single	0.009	0.007	0.006	0.023
19	Okpai	18	Onitsha	80	Double	0.0036	0.0272	0.024	0.0868
21	Afam	22	Alaoji	25	Double	0.009	0.007	0.006	0.023
22	Alaoji	18	Onitsha	138	Single	0.049	0.0419	0.035	0.125
23	Shiroro	3	Jebba TS	244	Double	0.0067	0.0702	0.062	0.22
23	Shiroro	24	Katampe	144	Double	0.0052	0.0401	0.0362	0.1318
23	Shiroro	25	Kaduna	96	Double	0.0034	0.0292	0.0245	0.0868
25	Kaduna	26	Kano	230	Single	0.009	0.068	0.058	0.28
25	Kaduna	27	Jos	197	Single	0.0081	0.0609	0.049	0.178
27	Jos	28	Gombe	265	Single	0.0095	0.081	0.067	0.24

### 2.2 Mathematical formulation of the problem

The power system in any electrical circuit is analysed by calculating the system voltages and/or currents under normal and abnormal scenarios [34]. However, the electrical quantity (current) is used in the analysis of this study because it provides insight into the contingency analysis, which is crucial in identifying potential weak spots in the network. The power loss evaluation's primary task is to determine the current value since the resistance value is constant and obtained from the transmission line parameter. Here unbalanced short circuits such as line-to-ground (SLGF), line-to-line (LLF), and double line-to-ground (DLGF) calculations analysis are developed and analysed on each phase of the three-phase network using the symmetrical component method.

### 2.3 Computation of unbalanced fault

The various unbalanced fault expressions are given in Eqs. (1), (2), (3), (4), (5), (6), (7), and (8).

1. Single Line to Ground Fault (SLGF) involves the positive sequence, negative sequence, and zero sequences. For a fault at bus K, a phase to ground, the symmetrical components of fault current are given in Eqs. (1) and (2).

(1) $${\text{Ι}}_{K}^{0}={\text{Ι}}_{K}^{1}={\text{Ι}}_{K}^{2}=\frac{V_{K}\left(0\right)}{Z_{kk}^{1}+Z_{KK}^{2}+Z_{kk}^{0}+3Z_{f}}$$

where $Z_{kk}^{1},\;Z_{KK}^{2}\;and\;Z_{kk}^{0}\;a\text{re the diagonal elemnets in the K axis}$ of which the corresponding bus impedance matrix and $V_{K}\left(0\right)\;\text{is the}\text{prefault voltage at K}.$

The fault phase current is

(2) $${\text{Ι}}_{K}^{abc}=A{\text{Ι}}_{K}^{012}$$

where A is the symmetrical component transformation matrix.

2. Line-to-Line Fault (LLF): involves two conductors exposed to each other through fault impedance$\;Z_{f\;}$ at a given bus K. The fault current equations are given in Eqs. (3) and (4) and the zero sequence is not required for this fault.

(3) $$\text{Current relationship}:{\text{Ι}}_{K}^{0}=0$$

(4) $${\text{Ι}}_{K}^{1}=-{\text{Ι}}_{K}^{2}=\frac{V_{K}\left(0\right)}{Z_{kk}^{1}+Z_{KK}^{2}+Z_{f}}$$

3. Double line-to-ground fault (DLGF): with this fault type, two-line conductors or phases come in contact both with each other through an impedance $Z_{f}$ and ground at given bus K. The symmetrical components of the fault current equations are as given in Eqs. (5), (6), (7), and (8)

(5) $${\text{Ι}}_{k}^{1}=\frac{V_{K}\left(0\right)}{Z_{K}^{1}+\frac{Z_{KK}^{2}\left(Z_{KK}^{0}+3Z_{K}\right)}{Z_{kk}^{1}+Z_{KK}^{2}+Z_{f}}}$$

(6) $${\text{Ι}}_{\text{Κ}}^{2}=\frac{V_{\text{Κ}}\left(0\right)-Z_{\text{ΚΚ}}^{1}{\text{Ι}}_{\text{ΚΚ}}^{1}}{Z_{\text{ΚΚ}}^{2}}$$

(7) $${\text{Ι}}_{\text{Κ}}^{0}=\frac{V_{\text{Κ}}\left(0\right)-Z_{\text{ΚΚ}}^{1}{\text{Ι}}_{\text{ΚΚ}}^{1}}{Z_{\text{ΚΚ}}^{0}+3Z_{f}}$$

The phase currents are obtained from Eq. (2), and the fault current is presented as in Eq. (8)

(8) $${\text{Ι}}_{\text{Κ}}\left(F\right)={\text{Ι}}_{\text{Κ}}^{\text{b}}+{\text{Ι}}_{\text{Κ}}^{\text{c}}$$

Using the sequence network components of the fault current, the symmetrical components of the ith bus voltages during fault are obtained as presented in Eqs. (9), (10), and (11) and the phase voltages during fault is given in Eq. (12).

(9) $$\;V_{i}^{0}\left(F\right)=0-Z_{kk}^{0}I_{k}^{0}$$

(10) $$V_{i}^{1}\left(F\right)=V_{\text{Κ}}\left(0\right)-Z_{kk}^{1}I_{k}^{1}$$

(11) $$\;V_{i}^{2}\left(F\right)=0-Z_{kk}^{2}I_{k}^{2}$$

where $V_{i}^{1}\left(0\right)=V_{i}\left(0\right)$ *is the prefault phase voltage at bus i*.

The phase voltages during fault are

(12) $${\text{V}}_{K}^{abc}=A{\text{V}}_{K}^{012}$$

The symmetrical components of fault current in line i to j are given as presented in Eqs. (13), (14), and (15).

(13) $${\text{Ι}}_{ij}^{0}\;=\frac{V_{i}^{0}\left(F\right)-V_{j}^{0}\left(F\right)}{Z_{ij}^{0}}$$

(14) $${\text{Ι}}_{ij}^{1}=\frac{V_{i}^{1}\left(F\right)-V_{j}^{1}\left(F\right)}{Z_{ij}^{1}}$$

(15) $${\text{Ι}}_{ij}^{2}=\frac{V_{i}^{2}\left(F\right)-V_{j}^{2}\left(F\right)}{Z_{ij}^{2}}$$

where $Z_{ij}^{0},\;Z_{ij\;}^{1}and\;Z_{ij}^{2}$ are the zero, positive, and negative-sequence components of the actual line impedance between buses j and I.

### 2.4 Line current injection network model

**Figure 2:**
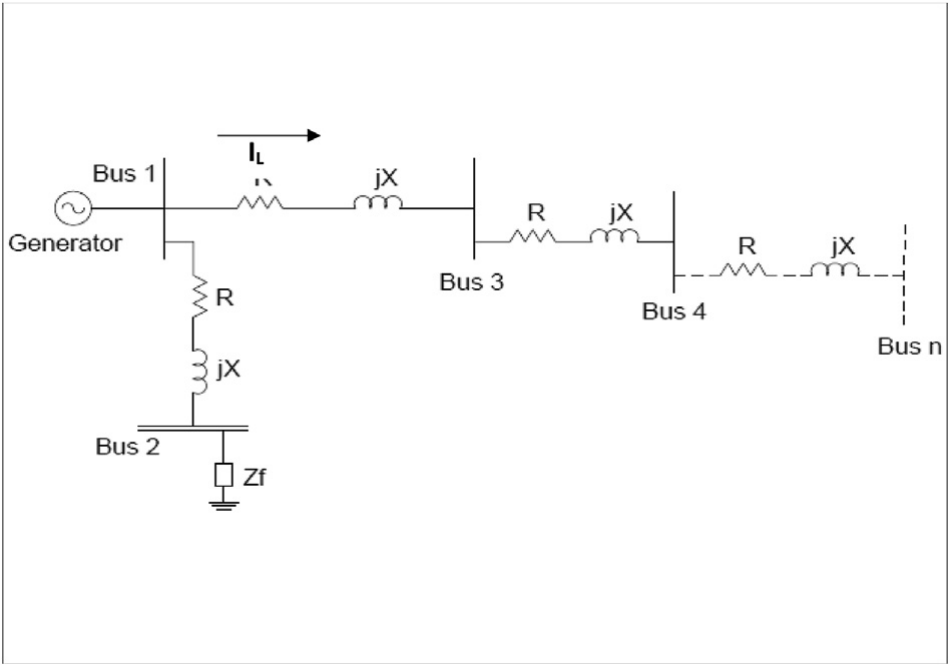
Line current network injection model.

The current dependent load model is used in which load changing is in direct proportion to current at constant voltage and constant power. Since current is required to flow in every branch of the network, the injection model of the current, shown in Figure 2, is constructed to flow in all power line branches with simulated fault at bus $K_{i}$ = 1, 2……. n.

**Figure 3:**
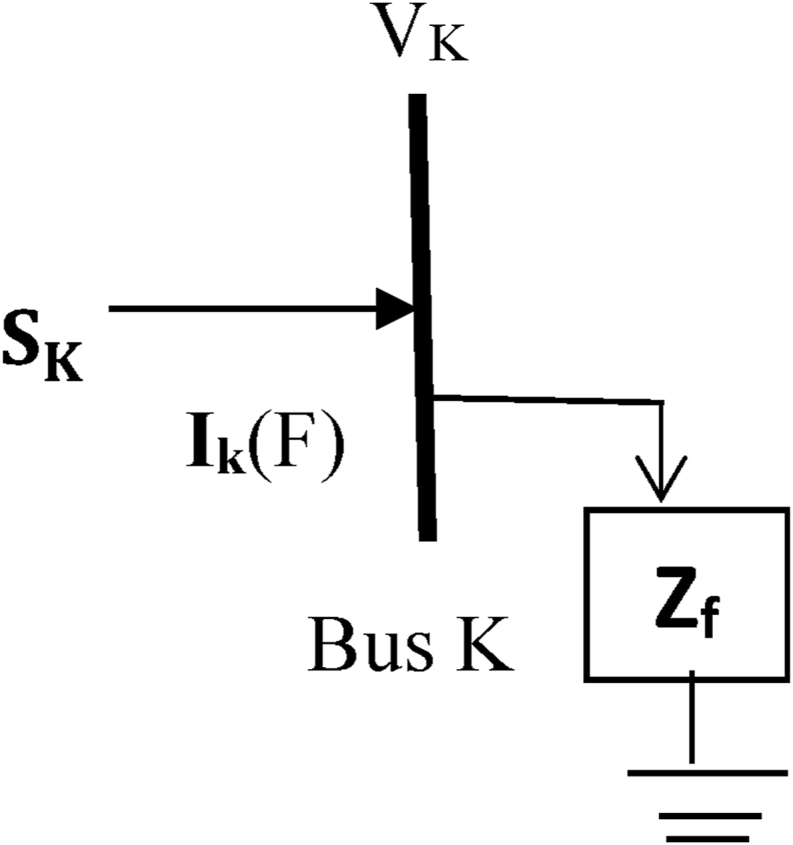
A typical bus K.

Therefore, Figure 3 represents a view of a typical fault simulated at bus K for a three-phase system;

The current flowing through the power line at any bus K is determined by Eq. (16)

(16) $${I^{*}}_{\text{K}\left(\text{F}\right)}=\frac{S_{K}}{\sqrt{3}V_{K}}=\frac{P_{K}+Q_{K}}{\sqrt{3}V_{K}}$$

If $I_{\text{K}}^{*}$ in Eq. (16) is decoupled into two separate parts it results into Eq. (17).

(17) $${I^{*}}_{K}=I_{K\left(real\right)}+I_{K\left(Imag\right)}$$

Hence, the real and reactive components of bus current in terms power and voltage buses are respectively given as

(18) $$I_{K\left(real\right)}=\frac{1}{\sqrt{3}\left|V_{k}\right|}\left(P_{K}cos\delta_{K}+Q_{K}sin\delta_{K}\right)$$

(19) $$I_{K\left(Imag\right)}=\frac{1}{\sqrt{3}\left|V_{k}\right|}\left(P_{K}sin\delta_{K}-Q_{K}\text{cos}\delta_{K}\right)$$

Using Eqs. (5), (6), (7), (8), (9), (10), (11), (12), (13), (14), and (15) and the mathematical model of Eqs. (16) and (17) to simulate the fault current in all the network nodes, then Eq. (16) can be rewritten as presented in Eq. (20).

(20) $$\overset{n}{\underset{k=1}{\sum }}{I^{*}}_{K}\left(F\right)=\frac{S_{K}}{V_{K}}$$

However, for this study, the line currents' results, obtained by using Eq. (20), are considered with the assumptions that the load and shunt reactance are neglected in the mathematical model. The premise is justifiable since positive and negative sequence currents are the same for static components like transformer and transmission lines [35, 36]. Besides, fault impedance 0.1 Ω is used; since the typical, expected value of the fault impedance in the 330-kV power line is between j0.1 and j0.3 Ω [37].

### 2.5 Fault simulation

**Figure 4:**
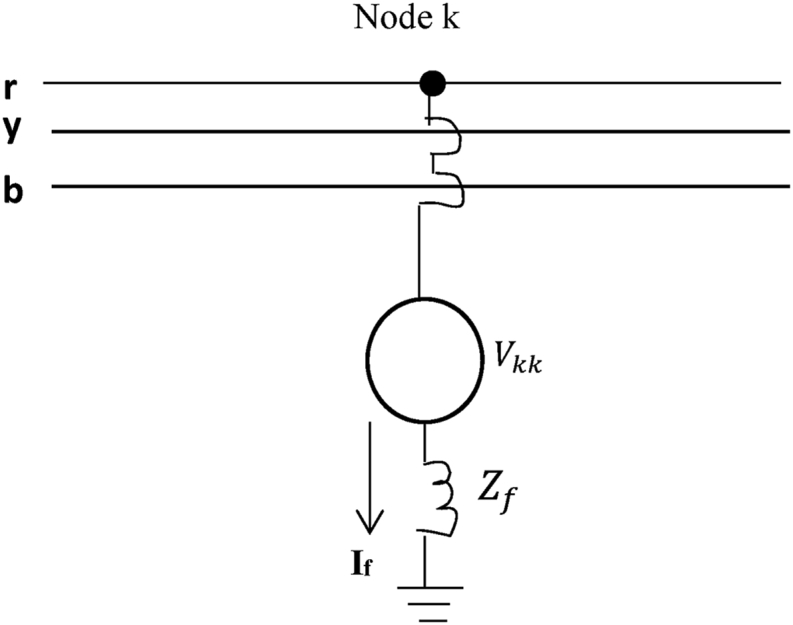
Model of a simulated fault.

By consider a three-phase transmission line with fault at node k, the model of a simulated fault is as shown in Figure 4. The fault at node k is simulated by switching on an impedance fault ($Z_{f}$) at bus k. and the new impedance ($Z_{kk(new)})$at bus k is given by Eq. (21).

(21) $$Z_{kk\left(new\right)}=Z_{bus}+Z_{f\;}$$

where $Z_{bus}$ is the bus impedance.

Thus, the fault current is given by Eq. (22):

(22) $$I_{f}\;=\frac{V_{kk}}{Z_{bus}+Z_{f}}$$

Because the system is complex, MATLAB software is employed to maintain the same computational flow chart. The MATLAB code starts by identifying the system input arguments in which variables are manly positive and zero sequence impedance, as presented in Table 1, to form the system impedance data. The simulation of various aspects of asymmetrical faults (for fault impedance,$\;Z_{f\;}$ = j0.1) is performed at every bus of the test system using an unbalanced fault algorithm written in a flexible MATLAB program environment. These faults, SLGF, LLF, and DLGF, are simulated at every bus of a three-phase power line network for the entire 28 buses and 33 branches of the test system.

## 3 Results analysis and discussions

**Figure 5:**
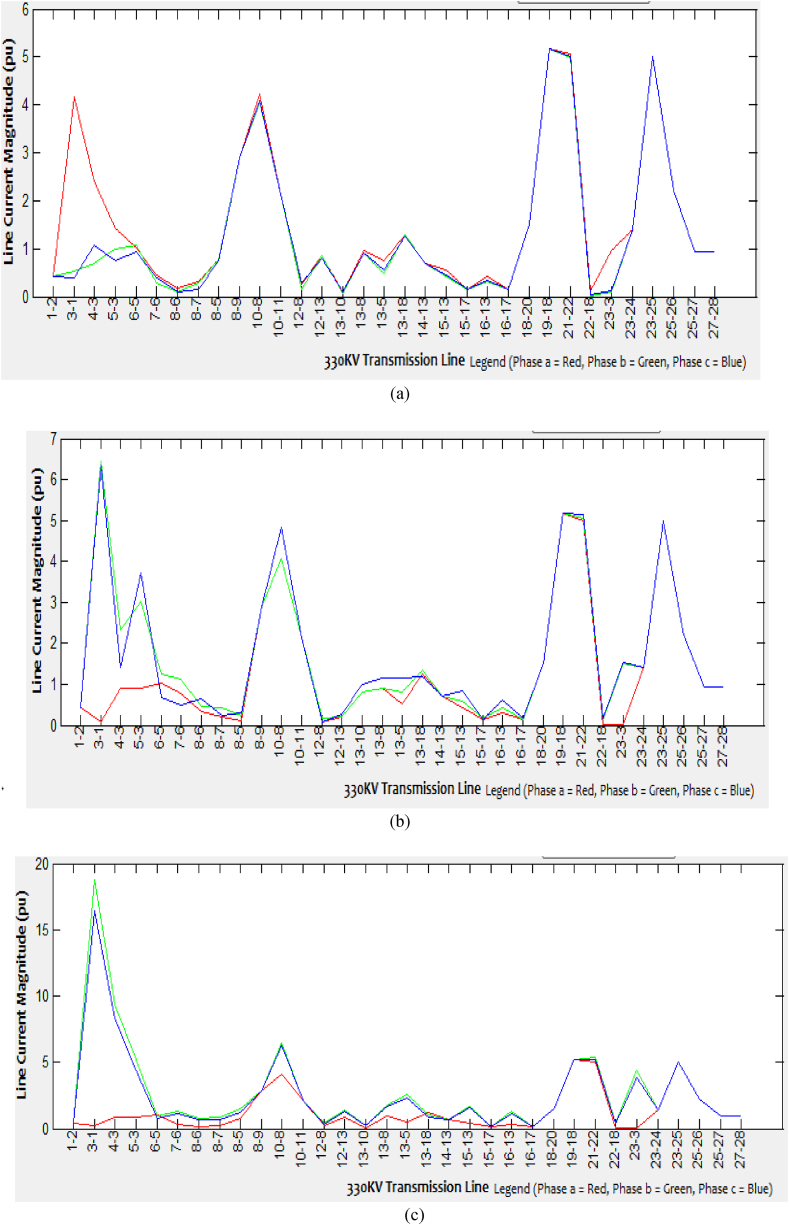
Line current magnitude of the (a) SLG faulted Kainji (bus 1), (b) L-L faulted Kainji (bus 1), (c) DLG faulted Kainji (bus 1).

**Figure 6:**
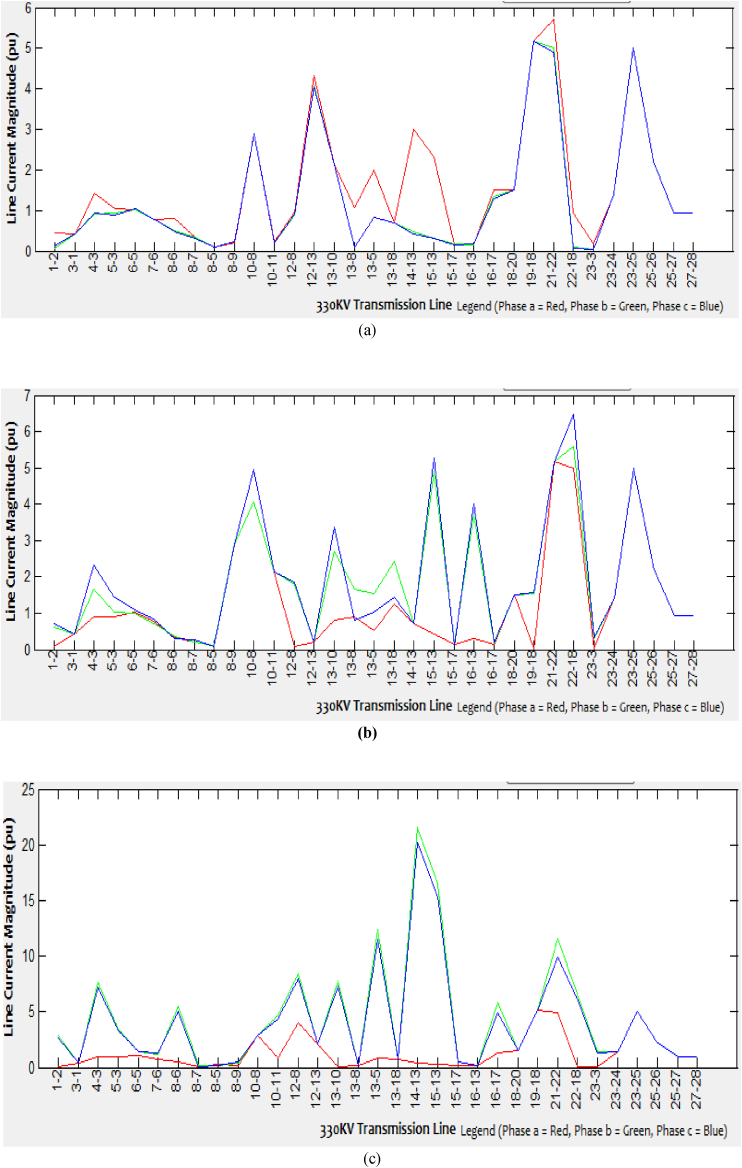
Line current magnitude of the (a) SLG faulted Benin (Bus 13), (b) L-L faulted Benin (Bus 13), (c) DLG faulted Benin (Bus 13).

**Figure 7:**
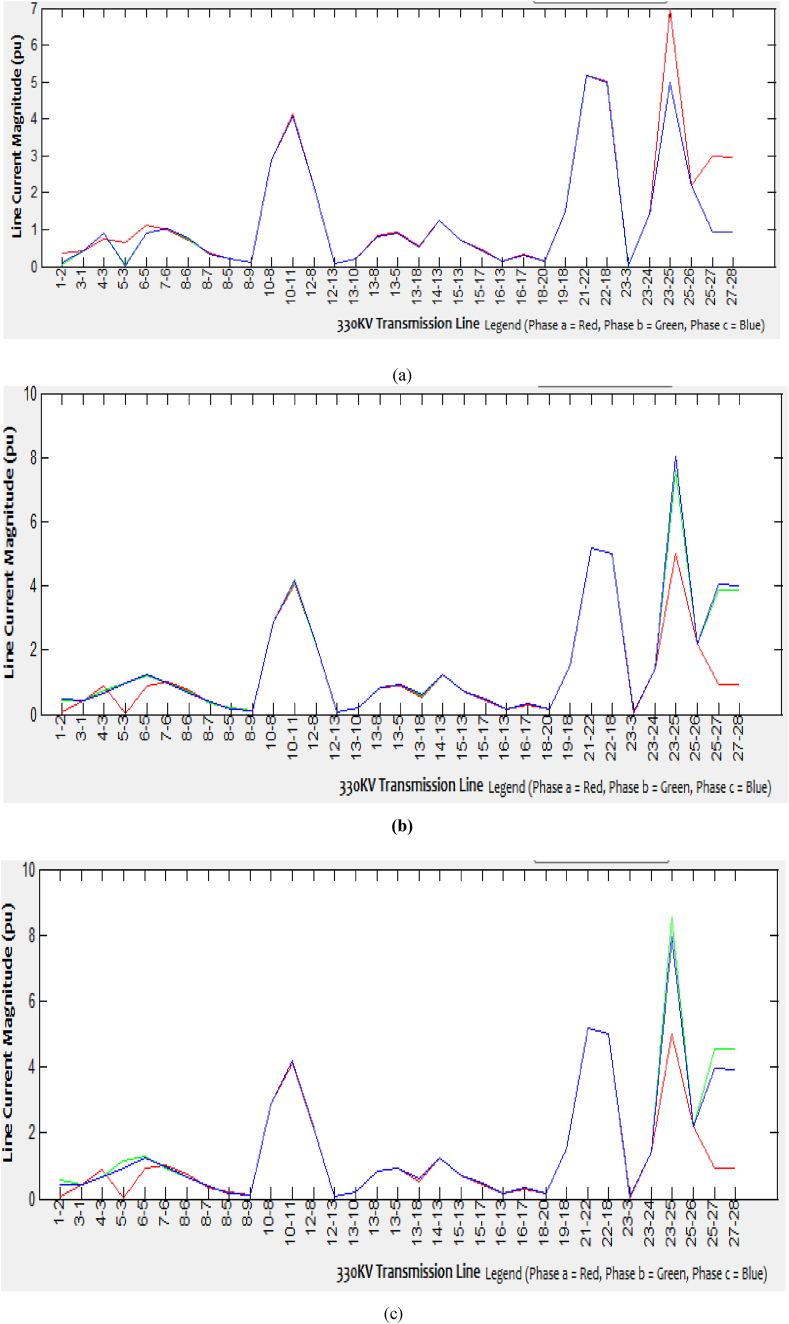
Line current magnitude of the (a) SLG faulted Gombe (Bus 28), (b) L-L faulted Gombe (Bus 28), (c) DLG faulted Gombe (Bus 28).

Various aspects of faulted bus simulations begin from Kainji bus one and end at Gombe bus twenty-eight for each of the 33-line currents are generated. Since the network is quite extensive, this paper only presents few samples of the faulted bus (Kainji bus 1, Benin bus 13, and Gombe bus 28) showing simulations for each of the 33 branches and 28 bus lines current as presented in Tables 2, 3, and 4. For each of the spectrum of line current obtained at every single faulted bus simulation of different types of unbalanced short-circuit faults into the three-phase system, the corresponding graphical representations are as shown in Figures Figure 5, Figure 6 and 7(a-c).

**Table 2:** Spectrum of line currents for faulted bus at Kainji (Bus 1) with Zf = j0.1.

From – To Bus	Line Current Magnitude (SLGF)			Line Current Magnitude (LLF)			Line Current Magnitude (DLGF)		
	Phase a (pu)	Phase b (pu)	Phase c (pu)	Phase a (pu)	Phase b (pu)	Phase c (pu)	Phase a (pu)	Phase b (pu)	Phase c (pu)
1-2	0.4217	0.4217	0.4217	0.4217	0.4217	0.4217	0.4217	0.4217	0.4217
1-3	4.162	0.52	0.3801	0.0807	6.439	6.3656	0.2314	18.7656	16.4299
4-3	2.4037	0.704	1.0632	0.8953	2.3195	1.4243	0.8927	9.3842	8.2384
5-3	1.4198	1.0004	0.745	0.9008	3.0357	3.7182	0.8648	5.1596	4.4045
5-6	1.0274	1.0853	0.9485	1.0245	1.2451	0.6681	1.013	0.9919	0.7578
7-6	0.4595	0.2771	0.3945	0.7639	1.1179	0.4824	0.3387	1.3437	1.1754
8-5	0.7758	0.7658	0.7494	0.0955	0.2372	0.3115	0.757	1.4778	1.2481
8-6	0.1899	0.0902	0.1053	0.3273	0.4473	0.6425	0.0981	0.7554	0.6805
8-7	0.3191	0.2758	0.1535	0.2185	0.4395	0.2249	0.2114	0.8723	0.6944
8-9	2.8835	2.8835	2.8835	2.8835	2.8835	2.8835	2.8835	2.8835	2.8835
10-8	4.2336	4.0692	4.0928	4.068	4.0559	4.83	4.0818	6.5076	6.2523
10-11	2.1338	2.1338	2.1338	2.1338	2.1338	2.1338	2.1338	2.1338	2.1338
12-8	0.2477	0.1431	0.2852	0.0899	0.1636	0.0829	0.2174	0.4275	0.3301
12-13	0.8483	0.8453	0.7958	0.2078	0.2077	0.2633	0.8193	1.3671	1.3424
13-10	0.1021	0.079	0.0964	0.8163	0.8149	0.9927	0.088	0.2613	0.2192
13-8	0.9555	0.9178	0.9004	0.904	0.9048	1.165	0.9087	1.7422	1.6875
13-5	0.7542	0.4732	0.5677	0.5077	0.812	1.1563	0.5227	2.5456	2.2951
13-18	1.2761	1.277	1.2557	1.2653	1.3434	1.2028	1.2658	1.0681	0.9048
14-13	0.7034	0.7034	0.7034	0.7034	0.7034	0.7034	0.7034	0.7034	0.7034
15-13	0.5676	0.4142	0.4604	0.4281	0.5893	0.8392	0.4384	1.7159	1.555
15-17	0.1561	0.1556	0.1577	0.1568	0.1585	0.1516	0.1567	0.1409	0.1393
16-13	0.4233	0.2993	0.3423	0.3135	0.4404	0.6277	0.3219	1.2997	1.1733
16-17	0.1574	0.1579	0.1558	0.1568	0.1553	0.1521	0.1568	0.1535	0.1546
18-20	1.4943	1.4943	1.4943	1.4943	1.4943	1.4943	1.4943	1.4943	1.4943
19–18	5.1746	5.1746	5.1746	5.1746	5.1746	5.1746	5.1746	5.1746	5.1746
21-22	5.0621	4.9879	5.0003	4.9992	5.0366	5.0357	4.9943	5.0109	5.0386
22-18	0.1168	0.02	0.035	0.031	0.1509	0.1712	0.0267	0.4456	0.3839
23-3	0.977	0.0891	0.1338	0.0284	1.4998	1.5256	0.0701	4.4233	3.8822
23-24	1.4096	1.4096	1.4096	1.4096	1.4096	1.4096	1.4096	1.4096	1.4096
23-25	5.0005	5.0005	5.0005	5.0005	5.0005	5.0005	5.0005	5.0005	5.0005
25-26	2.216	2.216	2.216	2.216	2.216	2.216	2.216	2.216	2.216
25-27	0.9441	0.9441	0.9441	0.9441	0.9441	0.9441	0.9441	0.9441	0.9441
27-28	0.9319	0.9319	0.9319	0.9319	0.9319	0.9319	0.9319	0.9319	0.9319

**Table 3:** Spectrum of line currents for faulted bus at Benin (Bus 13) when Z_f_ = j0.1.

From - To bus	Line Current Magnitude (SLGF)			Line Current Magnitude (L-LF)			Line Current Magnitude (DLGF)		
	Phase a (pU)	Phase b (pu)	Phase c (pu)	Phase a (pU)	Phase b (pu)	Phase c (pu)	Phase a (pU)	Phase b (pu)	Phase c (pu)
1-2	0.4217	0.4217	0.4217	0.4217	0.4217	0.4217	0.4217	0.4217	0.4217
1-3	0.4354	0.0638	0.1274	0.0807	0.6307	0.7046	0.0935	2.8641	2.6373
4-3	1.4309	0.9052	0.9254	0.8953	1.6584	2.3301	0.9153	7.6142	7.1521
5-3	1.0584	0.9234	0.8892	0.9008	1.0156	1.4385	0.9057	3.4887	3.3018
5-6	1.0208	1.0257	1.0342	1.0245	0.9903	1.0885	1.0301	1.3815	1.4103
7-6	0.7661	0.7714	0.7639	0.7639	0.7255	0.8451	0.7675	1.2164	1.2495
8-5	0.0987	0.0936	0.0948	0.0955	0.1167	0.078	0.323	0.2183	0.1273
8-6	0.8122	0.5077	0.4878	0.3273	0.3548	0.2942	0.4969	5.4342	5.0501
8-7	0.3347	0.3282	0.3183	0.2185	0.2026	0.2595	0.0942	0.1183	0.0762
8-9	0.217	0.218	0.2274	2.8835	2.8835	2.8835	0.2228	0.4514	0.4533
10-8	2.8835	2.8835	2.8835	4.068	4.0804	4.9506	2.8835	2.8835	2.8835
10-11	0.2211	0.21	0.1951	2.1338	2.1338	2.1338	0.8969	4.6718	4.371
12-8	0.9612	0.8824	0.9107	0.0899	1.7777	1.8599	4.0712	8.3932	7.9451
12-13	4.3169	4.0779	4.0649	0.2078	0.2122	0.2094	2.1338	2.1338	2.1338
13-10	2.1338	2.1338	2.1338	0.8163	2.7072	3.3743	0.0983	7.7994	7.2766
13-8	1.0725	0.0942	0.1038	0.904	1.6639	0.7965	0.202	0.212	0.2006
13-5	1.9913	0.8197	0.8339	0.5077	1.5283	1.0208	0.827	12.3477	11.5213
13-18	0.7034	0.7034	0.7034	1.2653	2.429	1.4277	0.7034	0.7034	0.7034
14-13	2.9981	0.4687	0.4081	0.7034	0.7034	0.7034	0.4371	21.5521	20.1436
15-13	2.3182	0.3085	0.3244	0.4281	4.8821	5.2611	0.3167	16.4651	15.3538
15-17	0.1683	0.1701	0.1466	0.1568	0.1894	0.1128	0.1579	0.468	0.4628
16-13	0.1508	0.1435	0.1675	0.3135	3.7272	4.0058	0.1559	0.2095	0.1952
16-17	1.5136	1.3309	1.302	0.1568	0.1589	0.22	1.314	5.8227	4.9082
18-20	1.4943	1.4943	1.4943	1.4943	1.4943	1.4943	1.4943	1.4943	1.4943
19–18	5.1746	5.1746	5.1746	0.031	1.5329	1.5557	5.1746	5.1746	5.1746
21-22	5.7101	5.0092	4.91	5.1746	5.1746	5.1746	4.9568	11.5746	9.9099
22-18	0.9277	0.1046	0.0724	4.9992	5.5859	6.484	0.0275	6.6108	6.1708
23-3	0.1796	0.0437	0.0276	0.0284	0.3134	0.34	0.0338	1.3941	1.3157
23-24	1.4096	1.4096	1.4096	1.4096	1.4096	1.4096	1.4096	1.4096	1.4096
23-25	5.0005	5.0005	5.0005	5.0005	5.0005	5.0005	5.0005	5.0005	5.0005
25-26	2.216	2.216	2.216	2.216	2.216	2.216	2.216	2.216	2.216
25-27	0.9441	0.9441	0.9441	0.9441	0.9441	0.9441	0.9441	0.9441	0.9441
27-28	0.9319	0.9319	0.9319	0.9319	0.9319	0.9319	0.9319	0.9319	0.9319

**Table 4:** Spectrum of line currents for faulted bus at Gombe (Bus 28) with Z_f_ = j0.1.

From - To bus	Line Current Magnitude (SLGF)			Line Current Magnitude (L-LF)			Line Current Magnitude (DLGF)		
	Phase a (pU)	Phase b (pu)	Phase c (pu)	Phase a (pU)	Phase b (pu)	Phase c (pu)	Phase a (pU)	Phase b (pu)	Phase c (pu)
1-2	0.4217	0.4217	0.4217	0.4217	0.4217	0.4217	0.4217	0.4217	0.4217
1-3	0.3555	0.05	0.0989	0.0807	0.4439	0.4632	0.0744	0.5462	0.438
4-3	1.1209	0.9118	0.905	0.9008	1.1996	1.2593	0.9104	1.3112	1.247
5-3	0.7601	0.8889	0.8881	0.8953	0.755	0.6452	0.8861	0.6472	0.6582
5-6	0.9822	1.0257	1.0216	1.0245	0.9779	0.952	1.0232	0.9469	0.9562
7-6	0.3547	0.3247	0.3296	0.3273	0.3581	0.3712	0.3273	0.3767	0.3681
8-5	0.7171	0.7581	0.7625	0.7639	0.7078	0.6745	0.7593	0.6739	0.678
8-6	0.1113	0.0969	0.0961	0.0955	0.1167	0.1244	0.0968	0.1263	0.1233
8-7	0.1923	0.221	0.2162	0.2185	0.1926	0.1748	0.2184	0.1718	0.1778
8-9	2.8835	2.8835	2.8835	2.8835	2.8835	2.8835	2.8835	2.8835	2.8835
10-8	4.1283	4.075	4.0708	4.068	4.1458	4.1822	4.0743	4.1865	4.1775
10-11	2.1338	2.1338	2.1338	2.1338	2.1338	2.1338	2.1338	2.1338	2.1338
12-8	0.2176	0.2016	0.2095	0.2078	0.2133	0.2162	0.2051	0.221	0.2144
12-13	0.828	0.8204	0.8166	0.8163	0.8341	0.8428	0.819	0.8419	0.8422
13-10	0.084	0.088	0.0898	0.0899	0.083	0.0754	0.0887	0.0779	0.0758
13-8	0.9234	0.9077	0.9047	0.914	0.9296	0.9238	0.9067	0.9233	0.9126
13-5	0.5576	0.5096	0.5107	0.5077	0.5701	0.5974	0.5109	0.6033	0.5932
13-18	1.2576	1.2646	1.2659	1.2653	1.26	1.2478	1.2653	1.252	1.2477
14-13	0.7034	0.7034	0.7034	0.7034	0.7034	0.7034	0.7034	0.7034	0.7034
15-13	0.4395	0.4297	0.4303	0.4281	0.4389	0.4247	0.4306	0.4393	0.4318
15-17	0.1564	0.1566	0.1568	0.1568	0.1562	0.1558	0.1567	0.156	0.1558
16-13	0.3178	0.3144	0.3153	0.3135	0.3246	0.3268	0.3153	0.3204	0.3145
16-17	0.1571	0.1569	0.1567	0.1568	0.1573	0.1577	0.1568	0.1575	0.1577
18-20	1.4943	1.4943	1.4943	1.4943	1.4943	1.4943	1.4943	1.4943	1.4943
19–18	5.1746	5.1746	5.1746	5.1746	5.1746	5.1746	5.1746	5.1746	5.1746
21-22	5.0106	4.9999	4.9991	4.9992	5.0152	5.0143	4.9996	5.0198	5.0137
22-18	0.0424	0.0317	0.0309	0.031	0.0475	0.0474	0.0313	0.0516	0.047
23-3	0.644	0.0466	0.0029	0.0284	0.991	0.9823	0.0225	1.1723	0.9411
23-24	1.4096	1.4096	1.4096	1.4096	1.4096	1.4096	1.4096	1.4096	1.4096
23-25	6.9909	5.0005	5.0005	5.0005	7.5876	8.038	5.0005	8.5379	7.9414
25-26	2.216	2.216	2.216	2.216	2.216	2.216	2.216	2.216	2.216
25-27	2.9857	0.9441	0.9441	0.9441	3.8743	4.0305	0.9441	4.5677	3.9438
27-28	2.9706	0.9319	0.9319	0.9319	3.8561	4.022	0.9319	4.552	3.9358

### 3.1 Unbalanced current circuit analysis

It has been discovered from the literature that most of the system collapses were a result of unbalance current in the three-phase when a fault occurs. Tables 2, 3, and 4 are the samples of the spectrum of line currents obtained when a numerical simulation of various aspects of faults on the three-phase power line was carried out begin from Kainji bus 1 and end at Gombe bus 28 for each of the 33-line currents. This study's analysis covers all the 33 lines for the simulated 28 buses and is categorized based on their characteristics, as revealed in this study into four scenarios. Scenario 1 is the line bus with the number of unbalanced current circuits ranging from 0 to 3, as shown in Table 5. Scenarios 2 is the number of unbalanced current circuits ranging from 10 to 12, as presented in Table 6. Scenarios 3 has the line bus with the number of unbalanced current circuits ranging from 18 to 21, as shown in Table 7. Scenarios 4 has either 27 or 28 unbalanced current circuits has revealed in Table 8.

**Table 5:** Scenarios 1 of line bus unbalance current circuit.

Line No.	Line Bus Name	Length (KM)	Circuit Type	Current unbalance circuit	Observation
l-2	Kainji-Birnin Kebbi	310	Single	None (0)	Current balanced with all the 28 faulted buses; this line will not trip on a 330kV grid with any faulted bus.
18–20	Onitsha-New Haven	96	Single	1	Current balanced except at faulted bus 23; trips 330kV line from Onitsha-New Haven
19–18	Okpai-Onitsha	80	Double	3	Current balanced except at faulted buses 13, 21, and 23; trips 330kV line from Okpai-Onitsha
23–24	Shiroro-Katampe	144	Double	1	Current balanced except at faulted bus 24; trips 330kV line from Shiroro-Katampe
23–25	Shiroro-Kaduna	96	Double	2	Current balanced except at faulted buses 26 and 27; these buses trips 330kV line from Shiroro-Kaduna
25–26	Kaduna-Kano	230	Single	None (0)	Current balanced with all the faulted buses; this line will not trip on a 330kV grid with any faulted bus.
25–27	Kaduna-jos	197	Single	2	Current balanced except at faulted buses 27 and 28; trips 330kV line from Kaduna-Jos
27–28	Jos-Gombe	265	Single	2	Current balanced except at faulted buses 27 and 28

**Table 6:** Case 2 of line bus with unbalance current circuit.

Line No.	Line Bus Name	Length (KM)	Circuit Type	Current unbalance circuit	Observation
8–9	Ikeja-Akangba	18	Double	12	Current unbalanced occur at faulted buses 9, 12, 13, 14, 15, 16, 17, 18, 19, 20, 21, and 22
10–8	Egbin-Ikeja West	62	Double	10	Current unbalanced occur at faulted buses 1, 4, 5, 6, 7, 8, 9, 10, 11, and 12
13–11	Egbin-Aja	28	Double	12	Current unbalanced occur at faulted buses 11, 12, 13, 14, 15, 16, 17, 18, 19, 20, 21, and 22
13–14	Benin-Ajaokuta	195	Double	10	Current unbalanced occur at faulted buses 6, 7, 8, 9, 10, 11, 12, 13, 14, 16, and 17

**Table 7:** Scenarios 3 of line bus with unbalance current circuit.

Line No.	Line Bus Name	Length (KM)	Circuit Type	Current unbalance circuit	Observation
5–9	Osogbo-Ayede	115	Single	20	Current balanced occur only at faulted buses 15, 16, 17, 18, 19, 20, 21, and 22
7–6	Papalanto-Ayede	60	Single	20	Current balanced occur only at faulted buses 15, 18, 19, 21, 22, 23, 25 and 26
8–5	Ikeja-Osogbo	235	Single	20	Current balanced occur only at faulted buses 15, 16, 17, 18, 19, 20, 21, and 22
8–7	Ikeja-Papalanto	30	Single	21	Current balanced occur only at faulted buses 15, 17, 18, 19, 20, 21, and 22
12–8	Omotosho-Ikeja	160	Single	21	Current balanced occur only at faulted buses 15, 23, 24, 25, 26, 27, and 28
12–13	Omotosho-Benin	120	Single	18	Current balanced occur only at faulted buses 2, 12, 14, 16, 17, 18, 19, 26, 27, and 28
13.18	Benin-Onitsha	137	Single	20	Current balanced occur only at faulted buses 2, 3, 23, 24, 25, 26, 27, and 28
15–17	Sapele Aladja	63	Single	19	Current balanced occur only at faulted buses 1, 2, 3, 23, 24, 25, 26, 27, and 28
16–17	Delta-Aladja	32	Single	19	Current balanced occur only at faulted buses 1, 2, 3, 23, 24, 25, 26, 27, and 28
21–22	Afam-Alaoji	25	Double	18	Current balanced occur only at faulted buses 1. 2, 21, 22, 23, 24, 25, 26, 27, and 28

**Table 8:** Scenario 4 of Line bus with unbalance current circuit.

Line No.	Line Bus Name	Length (KM)	Circuit Type	Current unbalance circuit	Observation
1–3	Kainji-Jebba (TS)	81	Double	28	No current balanced circuit.
4–3	Jebba (GS)-Jebba (TS)	8	Double	28	No current balanced circuit
23–3	Shiroro-Jebba TS	244	Double	28	No current balanced circuit
5–3	Osogbo-Jebba TS	157	3 x Single	28	No current balanced circuit
8–6	Ikeja-Ayede	137	Single	28	No current balanced circuit
13–5	Benin-Osogbo	251	Single	28	No current balanced circuit
10–13	Egbin-Benin	218	Single	28	No current balanced circuit
8–13	Ikeja-West-Benin	280	Double	27	No current balanced circuit except at the faulted bus 28
15–13	Sapele-Benin	50	Double	27	No current balanced circuit except at the faulted bus 28
16–13	Delta-Benin	107	Single	27	No current balanced circuit except at the faulted bus 28
22–18	Alaoji-Onistha	138	Single	27	No current balanced circuit except at the faulted bus 28

#### 3.1.1 Scenario 1

A large number of balanced currents characterizes the lines under this category. They are not affected by the faults occurring at different test system locations, as shown in Table 5. For instance, line 19-18 (Okpai-Onitsha) will trip on a 330kV grid when there is a fault at buses 13 (Benin), 21 (Afam), and 23 (Shiroro). Bus 13, Benin transmission station (TS) connected to Onitsha. In contrast, bus 19, Okpai, is a generating bus tied to other generating stations (i.e., Afam, Shiroro, Okpai, etc.) regarding the particular generation and scheduling features. Similarly, lines 23–24 (Shiroro-Katampe) and 23–25 (Shiroro-Kaduna) are affected by faulted buses at bus 24 (Shiroro) and bus 26 (Kano), respectively. Line 18–20 (Onitsha-New Haven) will trip on a 330kV grid when bus 23 (Shiroro) is faulted. In addition, lines 25–27 (Kaduna-Jos) and 27–28 (Jos-Gombe) shared some common features; the two lines are single circuits, and the buses that are directly connected to the faulted buses 27 and 28 affect these lines. The lines (1–2) and 25–26) have a current balance and will not trip on the 330kV grid with any of the faulted buses. It is also interesting to note that all the lines except lines 19-18 (Okpai-Onitsha) and 18–20 (Onitsha-New Haven) are from the Northern parts of the grid.

#### 3.1.2 Scenario 2

In this category, the unbalanced current circuit ranges from 10 to12 in number, is associated with a double circuit, as presented in Table 6. These lines, except line 13–14 (Benin to Ajaokuta), are also short lengths (∠80km), and the effect of unbalanced faults from different locations on these lines are fair, as shown in Table 6.

#### 3.1.3 Scenario 3

These lines have a particular shared characteristic of a single circuit except line 21–22 (Afam-Alaoji) with double circuits. There are ten (10) lines in this category, having a significant unbalance current ranging from 18 to 21 in numbers. However, the five lines, i.e., 12–13 (Omotosho-Benin), 13–18 (Benin-Onitsha), 15–17 (Sapele Aladja), 16–17(Delta-Aladja), and 21–22 (Afam-Alaoji) from the eastern part will not trip on 330kV grid with any of these northern faulted buses, 26 (Kano), 27 (Jos) and 28 Gombe) as presented in Table 7.

#### 3.1.4 Scenario 4

The eleven (11) lines in this category are the worst in unbalanced current circuits on the 330kV grid. When a short circuit occurs, all the faulted 28 buses affect these lines. These are the most critical lines when a fault occurs because only the faulted bus 28 has a balanced current circuit in all the three phases of the different unbalanced faults, as shown in Table 9. Six (6) out of these lines are single circuit with medium length (between 80km and 250km) while the remaining five (5) lines are double circuit with both short lengths (∠80km) and medium lengths, as shown in Table 8.

**Table 9:** Calculated power line loss based on maximum line current.

From	To	Max. line current (I) (pu)	I pu X 0.175 (kA)	$I^{2}$(kA)	R (pu)	R. (pu) X 1089 (ohm)	Power Line loss $I^{2}R$(MW)	Scenario No (unbalanced current)
5	8	23.9129	4.1848	17.5125	0.0049	5.3361	**93.4468**	Scenario 3
13	18	22.6256	3.9595	15.6776	0.0049	5.3361	**83.6566**	Scenario 3
15	17	24.0364	4.2064	17.6938	0.0023	2.5047	**44.3170**	Scenario 3
5	6	11.4695	2.0072	4.02885	0.0041	4.4649	**17.9877**	Scenario 3
21	22	9.8982	1.2072	1.4573	0.0090	9.8010	**16.9772**	Scenario 3
18	22	25.4175	4.4481	19.7856	0.0049	5.3361	**105.5761**	Scenario 4
5	13	12.3477	2.1608	4.66906	0.0089	9.6921	**45.2550**	Scenario 4
13	16	21.9803	3.8466	14.7963	0.0023	2.5047	**37.0595**	Scenario 4
13	14	10.7761	1.8858	3.5562	0.0070	7.6230	**27.1095**	Scenario 2
18	20	15.7524	2.7567	7.5994	0.0036	3.9204	**29.7920**	Scenario 2
25	27	7.6950	1.3466	1.8133	0.0081	8.8209	**15.9958**	Scenario 2
1	2	8.9730	1.5703	2.4658	0.0111	12.0879	**29.8059**	Scenario 1
25	26	7.7021	1.3479	1.8168	0.0090	9.8010	**17.8059**	Scenario 1

To emphasize the importance of the calculated line loss are indicated in bold values.

### 3.2 Analysis of power loss on the line

**Figure 8:**
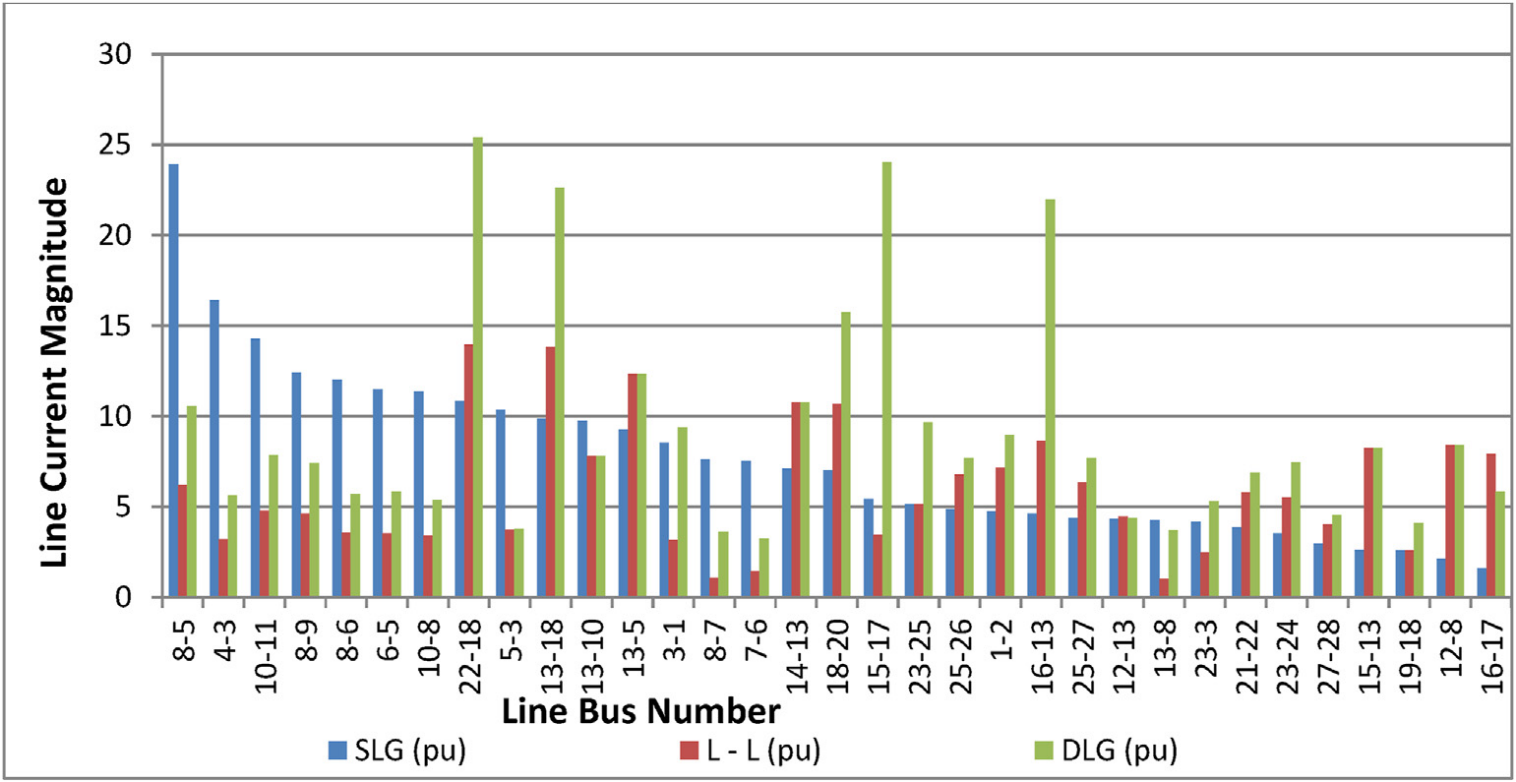
Maximum line current obtained for SLG, L-L, and DLG when Z_f_ = j0.1.

To achieve higher efficiency and stability in the system, it became necessary to unveil lines that experience high power loss in this study based on the result of the unbalanced current analysis. The results of all the line current magnitudes obtained (samples are shown in Tables 2, 3, and 4) during simulation of unbalance fault in the test system are analysed or streamlined to establish a categorical data of maximum line current magnitudes rigorously. Thus, the possible peak current level found in Figure 8 is used to calculate the expected available maximum range of power losses that can occur on each of the critical lines under study, as presented in Table 9.

It becomes crystal clear from the power line loss analysis of Table 9 that most of the lines with high magnitudes of unbalanced current also have a high value of power line losses. These lines include most of the lines discussed under Scenario 3 and 4 of Tables 7 and 8, respectively. For instance, in Table 7, the established unbalanced current, lines 8-5 (Ikeja-Osogbo), 13–18 (Benin-Onitsha), and 16–17 (Sapele-Aladja) lines having significant power line losses of 93.5MW, 83. 7MW, and 44.3MW, respectively, as shown in Table 9. Such sequences are marked as “critical lines” in this study and require urgent attention. Other critical lines in this category are lines 5–6 (Osogbo-Ayede) and 21–22 (Afam-Alaoji), with high power line losses of 18MW and 17MW, respectively.

Also, in Table 8 (scenario 4), except lines with a double circuit that have a reasonable value of line losses, all the single circuit lines, like lines 22-18 (Alaoji-Onistha) and 13-5 (Benin-Osogbo), and 16-13 (Delta-Benin) are critical lines having losses of 105.6MW, 45.3MW, and 37.06MW respectively. It is worth discussing that line 22-18 (Alaoji-Onistha) has the highest power line losses among all the 33 lines analysed. From Table 6 (i.e., scenario 2), the unbalance current of line 13–14 (Benin to Ajaokuta) is a critical path with the highest line loss value of 27.1MW in that category, as shown in Table 9.

With a good understanding of the result of Table 5 (scenario 1), characterized by a large number of balanced currents when faults occur at different locations, the power line loss as revealed in Table 9 is also reasonable, corresponding to its low current magnitude as reflected. It is also interesting to note that majority of the lines in this category are with double circuits. For instance, line 9–18 (Okpai-Onitsha) is a double circuit with a considerable power line loss of 5MW compare to single circuit lines 18–20 (Onitsha-New Haven) and 25–27 (Kaduna-Jos), characterized by a high line loss of 29.8MW and 15.9MW respectively, as presented in Table 9. It is worth mentioning here that lines 1–2 (Kainji-Birnin Kebbi) and 25–26 (Kaduna-Kano), single circuits, and long lines with high line loss threats 29.5MW and 18MW, respectively, display the lowest magnitude level of balanced current as shown in Table 6.

#### 3.2.1 Proposed 330kV quad bundle conductors design of transmission line

From the analysis of unbalance current circuit and power line losses carried out in this study, it is found that significant current unbalance and line losses are discovered for both single circuit (SC) and double circuit (DC) on the network. A careful study of these critical lines showed that these lines need ‘upgrade’ or ‘conversion’ as the case may be. It should be noted here that most of the existing double circuits are double conductor bison. The proposed specifications required for the construction of quad bundle conductors are presented in Eqs. (23), (24), (25), (26), (27), (28), and (29), and the generated result for the established critical lines is shown in Table 10 to improve the efficiency and power transfer capability of the Nigerian transmission lines network.

**Table 10:** Critical DC and SC lines upgraded characteristics.

Bus no/Bus name		Bus no/Bus name		Length	R (Ω/km)		L (mH/km)		X_L_ (Ω/km)		C (μF/km)	
From		To		(km)								
22	Alaoji	18	Onitsha	138		2.07966		121.7022		38.2398		18.4506
8	Ikeja-West	5	Oshogbo	235		3.54145		207.2465		38.2398		31.4195
13	Benin	18	Onitsha	137		2.06459		120.8203		37.9627		18.3169
13	Benin	5	Oshogbo	251		3.78257		221.3569		69.5521		33.5587
15	Sapele	17	Aladja	63		0.94941		55.5597		17.4573		8.4231
16	Delta	13	Benin	107		1.61249		94.3633		29.6497		14.3059
13	Benin	14	Ajaokuta	195		2.93865		171.9705		54.0345		26.0715
10	Egbin	13	Benin	218		3.28526		192.2542		60.4078		29.1466
18	Onitsha	20	New Haven	96		1.44672		84.6624		26.6016		12.8352
8	Ikeja	6	Ayede	137		2.06459		120.8203		37.9627		18.3169
5	Osogbo	6	Ayede	115		1.73305		101.4185		31.8665		15.3755
10	Egbin	11	Aja	14		0.21098		101.4185		3.8794		1.8718
21	Afam	22	Alaoji	25		0.37675		12.3466		6.9275		3.3425
25	Kaduna	26	Kano	230		3.4661		c2∗a16		63.733		30.751
25	Kaduna	27	Jos	197		2.96879		202.837		54.5887		26.3389

The resistance (RT) of the four-conductor bundle is calculated using Eq. (23).

(23) $$R_{T}=\frac{\rho L}{A}$$

ρ=Resistivity of Aluminum;

A=Cross sectional Area of Aluminum in the bundle; and

L=Length of the line (1 km).

On substituting, we have (for one conductor)

$$R_{T}=\frac{2.8735\times 10^{-8}\text{ Ω}m\times 1000m}{476.6\times 10^{-6}m^{2}\times 4}$$

$$R_{T}=0.01507\;\Omega /km$$

**Resistance (R) =0.01507 Ω/km** for four bundle conductors.

The Geometric Mean Radius (GMR) and GMD are given in Eqs. (24) and (25) respectively.

(24) Geometric Mean Radius (GMR) = 1.091 ∜ (r. dˆ3)

$$GMR=1.091\sqrt[{4}]{0.015075\times 0.646^{3}}$$

GMR=0.2755m

(25) $$GMD=\sqrt[{3}]{D_{AB}\times D_{BC}\times D_{AC}}$$

$$GMD=\sqrt[{3}]{14\times 14\times 28}$$

GMD=17.64m

System of conductors is 14m while the bundle diameter for a four-bundle conductor arrangement is 64.6 cm. The radius of the camel conductor is 0,015075m.where:

r= Radius of the conductor;

d=Bundle diameter; and

$D_{AB},D_{BC},D_{AC}=$Distance between phase conductors.

The Line Inductance (L) and Inductive Reactance (X_L_) are calculated using Eqs. (26) and (27) respectively.

(26) L=2×10−7lnGMDe−14GMR

L=2×10−7ln17.64e−14×0.2755×1000m

L=0.8819mH/km

(27) $$\;X_{L}=2\pi fL$$

$$X_{L}=2\pi \times 50\times 0.8819\times 10^{-6}$$

$$X_{L}=0.2771\;\Omega /\mathrm{km}$$

The Capacitance is calculated using equation (28):

(28) $$C=\frac{2\pi \varepsilon_{o}}{\text{ln}\left(GMD/GMR\right)}$$

$$C=\frac{2\pi \times 8.85\times 10^{-12}}{\text{ln}\left(17.64/0.2755\right)}$$

C=0.01337μF/km

The line Susceptance is calculated using Eq. (29).

(29) B=2πfC

$$B=2\times 3.142\times 50\times 0.01337\times 10^{-6}$$

$$B=4.2\times 10^{-6}Siemens/km$$

Thus, the generated electrical line parameters (R, L, $X_{L}\;,\;C$ ) of the DC 330 kV for the critical lines are summarized in Table 10.

The phenomenon of reconductoring to Quad-bundle conductors for those critical lines presented in Table 10 has already been established during the study's analysis. Thus, the design of Table 10 can be implemented accordingly. Moreover, this analysis is performed under the abnormal condition for both unbalanced current and power line losses; hence, the 330kV grid system in which these lines are embedded can become increasingly unstable during any electrical disturbances. Also, for every long line, the power flow is limited to the line's surge impedance loading, which is already considered in this design. Meanwhile, most of these lines are single circuits and characterized by many unbalanced circuits and high-power line losses, as analysed in Tables 5, 6, 7, and 8 and Table 9. For instance, the single circuit lines: 22-18 (Alaoji-Onitsha); 8-5 (Ikeja-West-Osogbo); 13–18 (Benin-Onitsha); 13-5 (Benin-Osogbo); 15–17 (Sapele-Aladja); and 16-13 (Delta- Benin) display significant current unbalance and contributing significantly to power line losses. The transmission stations line 22-18 (Alaodji-Onitsha) recorded the highest value of power line losses, and Ikeja West-Osogbo 330kV transmission substations closely follow this. Also, the separating distances between the conductors on the transmission lines with double-circuit and unbalanced current lines are recorded in Benin-Ajaokuta, Egbin-Aja Afam-Alaodji are upgraded, as shown in Table 10.

#### 3.3 Recommendations to improve the test system

Based on this study's significant findings, the following suggestions are considered a desirable approach to enhance sound power transmission and system security during a disturbance on the Nigerian 330kV grid network. These recommendations are urgent and need to be implemented alongside the generating stations' increase capacity plan.

• Distance relay protection improvement of the most relatively critical unbalance current circuit lines identified during the disturbances at various locations on the network is urgently required to prevent these line circuits' constant tripping. These lines include Ikeja West-Osogbo, Benin-Osogbo, Alaoji-Onitsha, Delta-Benin, Egbin-Benin, Benin-Onitsha, Osogbo-Ayede, Sapele-Aladja, and Afam-Alaoji. It was even reported in the literature review that Afam-Alaoji tripped nine (9) times on fault between 6/5/2015 and 1/9/2017, which is clear evidence of inadequate protection relaying scheme. It should also be noted that these lines are connected to either the generator bus or Osogbo bus (National Control Centre), or Benin bus (links the Eastern, Western, and Northern parts of the network), except Alaoji-Onitsha. Thus, these lines will collapse in the event of significant fault occurrence without adequate protection relaying scheme.
• It is discovered from Table 5 in this study that the majority of the lines from the Northern parts of the grid have current balance circuits and will not trip on the 330kV grid with any of the faulted buses. That way, there is an urgent need to introduce multiple loops and additional lines in the network to form a ring circuit, especially the much-needed link between the Southern and Northern parts of the grid. Thus, the Nigerian Transmission Company Management should intensify significant projects (North-West ring, North-East ring, and Nambilla evacuation transmission lines). The project will form part of the network ring with Kainji-Birnin Kebbi, and Kaduna-Kano will guarantee continuity of supply in the event of any contingency or fault.
• Further investigation in the study revealed that additional lines are required from Shiroro, Benin, Omotosho substations, and the modification of these lines to form part of North West ting. A similar transformation can be implemented from Gombe, Kano, and a new substation (links Gombe) to form part of the North East ring.
• A new substation should be introduced at Kangiwa (distance calculator used), about 189 KM and 122 KM away from Kainji and Birnin Kebbi. It is worth mentioning here that line 1–2 (Kainji-Birnin Kebbi), a single circuit and long line that is not limited by stability limits, has the lowest magnitude level of the balanced current circuit as presented in Table 6. However, this line is among the lines with high power line losses, causing load curtailment and limiting the load levels. The 1–2 (Kainji-Birnin Kebbi) is not considered for conversion to double circuits in this study because electromagnetic coupling lines are more pronounced on double circuit towers with longer lengths (310 KM).
• There is an urgent request to replace those identified critical lines with the newly designed specifications for quad bundle conductors, as shown in Table 10, to enhance the network efficiency and power transfer capability. These lines are over-aged and characterized by high losses coupled with an increased number of unbalanced current circuits during the fault, as shown in Tables 7, 8, and 9. Moreover, the upgrade of the double circuits' critical lines and the single circuit essential lines' conversion to the four bundle conductors designed in Table 10 is also in anticipation of the transmission line expansion up to 2030. In line with this, a 330 kV line with the double circuit is proposed with 4-bundle (Quad) Bison conductors for each circuit as designed.
• The transmission grid may also need to be made more ‘intelligent’ through the configuration and training of Artificial Neural Network (ANN). That way, applying a set of line currents obtained from the numerical simulation of various aspects of faults (samples are shown in Tables 3, 4, and 5) can serve as input data. That produces the desired output set by providing input and matching output patterns in a supervised learning paradigm. For instance, the rigorous data generated in this study for the entire thirty-three (33) line currents in the existing network can be used to train ANN and valuable for system expansion planning.

## 4 Conclusion

This study developed a novel technique that provides insight into the current quantity occurring in the thirty-three (33) branches of the Nigerian 330kV transmission network when subjected to various aspects of asymmetrical faults at twenty-eight (28 buses) different locations. An ideal algorithm was developed for a set of unbalanced short-circuit calculations models of the network. It simulated the unbalanced short-circuit faults (SLG, LL, DLG) into each line of three-phase networks. The novel approach was implemented to form the basis to develop a strategy that identified the network's different problem areas, such as current unbalance circuit, high line current magnitude and losses, and critical weak lines. Based on these findings, the lines revealed that enough provision was not provided to have a close loop in the system. The study moved further to explore calculation and design parameters for the critical lines that require urgent attention as a power grid strengthening strategy. The study unveiled the Nigerian 330kV national grid's terrible and unsatisfactory state and proffered recommendations that need modifications to promote better efficiency, reliability, and security in the power line network. It is evident from the study that the priory of the transmission line expansion plan is of more priority over an increase in generation capacity for the evacuation of the generated energy. The data generated in this study will help system expansion planning, utilities desirous of minimizing network losses, and policymakers wishing to formulate more effective policies.

## Declarations

### Author contribution statement

Ademola Abdulkareem: Conceived and designed the experiments; Performed the experiments; Analyzed and interpreted the data; Wrote the paper.

A. Adesanya: Performed the experiments; Wrote the paper.

A. F. Agbetuyi & A. S. Alayande: Analyzed and interpreted the data; Contributed reagents, materials, analysis tools or data; Wrote the paper.

### Funding statement

This work was supported by the 10.13039/501100012497Covenant University Centre for Research, Innovation and Discovery.

### Data availability statement

Data will be made available on request.

### Declaration of interests statement

The authors declare no conflict of interest.

### Additional information

No additional information is available for this paper.
